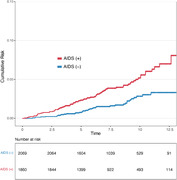# Association of Acquired Immunodeficiency Syndrome with Alzheimer`s Disease‐Related Dementia in HIV‐Infected Individuals

**DOI:** 10.1002/alz.084075

**Published:** 2025-01-09

**Authors:** Young‐gun Lee, Eunyoung Lee, Jihwan Bang

**Affiliations:** ^1^ Ilsan Paik Hospital, Inje University College of Meidicine, Goyang, Gyeonggi Korea, Republic of (South); ^2^ Boramae Medical Center, Seoul, Dongjak Korea, Republic of (South)

## Abstract

**Background:**

Cognitive decline often follows human immunodeficiency virus (HIV) infection, yet the specific risk factors for developing Alzheimer’s disease and related dementia (ADRD) in HIV patients remain elusive.

**Method:**

To investigate the association between acquired immune deficiency syndrome (AIDS) status at the time of HIV diagnosis and the risk of ADRD, we conducted a retrospective cohort study using data from a nationwide claim database spanning 2008 to 2021. During the study period, 13,289 patients were newly diagnosed with HIV infection and were prescribed antiretroviral therapy (ART). Among them, a total of 3,929 individuals over 40 years old, with a follow‐up exceeding 3 years and without dementia diagnosed within two months post‐HIV diagnosis, were included. Cox proportional hazards regression models for ADRD requiring medication such as anti‐cholinesterase inhibitor or memantine were performed using baseline presence of AIDS as a predictor, after adjusting for age, sex, insurance type, and comorbidities.

**Result:**

Among the 3,929 participants (median age: 45 [IQR 15] years; 90.9% male), with a median follow‐up of 7.6 (IQR 5.0) years, 114 patients were diagnosed with ADRD. HIV individuals with AIDS at baseline had a higher risk of ADRD (hazard ratio [HR], 2.21; 95% confidence interval [CI], 1.51 – 3.31) compared to those without AIDS. The risk was more prominent in individuals under 50 years old (HR, 5.39; 95% CI, 2.22 – 13.05) than in those 50 years or older (HR, 1.68; 95% CI, 1.07 – 2.63; *P* for interaction = 0.03).

**Conclusion:**

This study underscores the need to stratify the management of cognitive impairment in newly diagnosed HIV patients based on their baseline AIDS status and age.